# Posterior Reversible Encephalopathy Syndrome in Guillain-Barré Syndrome: Just a Problem of Immunoglobulins? Controversy From Two Atypical Case Reports

**DOI:** 10.3389/fneur.2022.817295

**Published:** 2022-04-06

**Authors:** Enrico Belgrado, Ilaria Del Negro, Daniele Bagatto, Simone Lorenzut, Giovanni Merlino, Gian L. Gigli, Mariarosaria Valente

**Affiliations:** ^1^Neurology Unit, Azienda Sanitaria Universitaria Friuli Centrale, Udine University Hospital, Udine, Italy; ^2^Clinical Neurology Unit, Azienda Sanitaria Universitaria Friuli Centrale, Udine University Hospital, Udine, Italy; ^3^Department of Medicine (DAME), University of Udine, Udine, Italy; ^4^Neuroradiology Unit, Department of Imaging, Azienda Sanitaria Universitaria Friuli Centrale, Udine University Hospital, Udine, Italy

**Keywords:** posterior reversible encephalopathy syndrome, neuroimaging, immunoglobulins, clinical neurology, endothelial dysfunction

## Abstract

**Background:**

Posterior reversible encephalopathy syndrome (PRES), reversible cerebral vasoconstriction syndrome (RCVS), or the coexistence of these two entities shares similar risk factors and clinical features. For these conditions, a common origin has been supposed. Even if the majority of patients show a favorable course and a good prognosis, a small percentage of cases develop neurological complications. Up to date, only about 30 cases of PRES associated with Guillain-Barré syndrome (GBS) have been reported in the literature.

**Cases:**

Here, we present two cases of a particularly aggressive PRES/RCVS overlap syndrome, associated with acute motor axonal neuropathy (AMAN) and acute inflammatory demyelinating polyneuropathy (AIDP) variants of GBS, respectively, presenting with similar initial clinical aspects and developing both an atypical and unfavorable outcome. On MRI examination, the first patient showed typical aspects of PRES, while, in the second case, radiological features were atypical and characterized by diffusion restriction on the apparent diffusion coefficient (ADC) map. The first patient demonstrated rapid worsening of clinical conditions until death; the second one manifested and maintained neurological deficits with a permanent disability.

**Conclusions:**

We suggest that PRES may conceal RCVS aspects, especially in most severe cases or when associated with a dysimmune syndrome in which autoimmune system and endothelial dysfunction probably play a prominent role in the pathogenesis. Although the role of IVIg treatment in the pathogenesis of PRES has been proposed, we suggest that GBS itself should be considered an independent risk factor in developing PRES.

## Introduction

Posterior reversible encephalopathy syndrome (PRES) is a clinical-radiological entity characterized by a potentially reversible subcortical vasogenic brain edema and acute neurological symptoms (1).

Reversible cerebral vasoconstriction syndrome (RCVS) is a group of clinical entities characterized by prolonged, reversible vasoconstriction of the cerebral arteries that resolve within 3 months, associated with a rapid or gradual onset of severe headache, and complicated by other neurological symptoms (stroke, hemorrhage, encephalopathy, concomitant PRES, and seizures) in up to one-third of patients (2).

The pathophysiology of PRES, RCVS, or their rarer co-existing syndrome is still a matter of debate. In light of similar risk factors and clinical features, a common origin has been supposed.

In most patients, a prognosis is generally favorable due to the reversibility of those syndromes; however, permanent disability or even death can be found in about 3–10% of cases (3).

## Case Description

### Patient 1

A 45-year-old woman was referred to the emergency room (ER) with a 4-day history of leg paresthesia. Two weeks before, she had a gastroenteritis-like illness. The remaining personal history was unremarkable. Neurological examination revealed walk impairment, reduction in pinprick sensation and sense of position in the distal part of legs, diffuse absence of osteotendinous reflexes, mild reduction of strength, cranial nerves were spared, and no sphincter dysfunction was present. Blood pressure (BP), heart rate (HR), and other vital signs were within normal range. The CSF examination showed high proteins (1,7 gr/L) with a normal leukocytes count.

A neurophysiological examination was performed, including motor nerve conduction studies, F-waves evaluation (Median, Ulnar, SPE, and SPI nerves bilaterally), and sensory nerve conduction studies (sural, superficial radial, median, and ulnar nerves bilaterally), leading to the diagnosis of an acute motor axonal neuropathy (AMAN) variant of Guillain-Barré syndrome (GBS) (4).

Immunomodulatory therapy with intravenous immunoglobulins (IVIg) was started (0.4 g/Kg/die for 5 consecutive days); however, 12 h after terminating the first infusion of IVIg, the patient complained of blurred vision and headache, soon after a convulsive seizure occurred. Continuous monitoring of BP and HR in the stroke unit did not reveal any alteration preceding the events.

The electroencephalogram showed multifocal epileptiform discharges mainly in the central and posterior regions.

**Figure 1:**
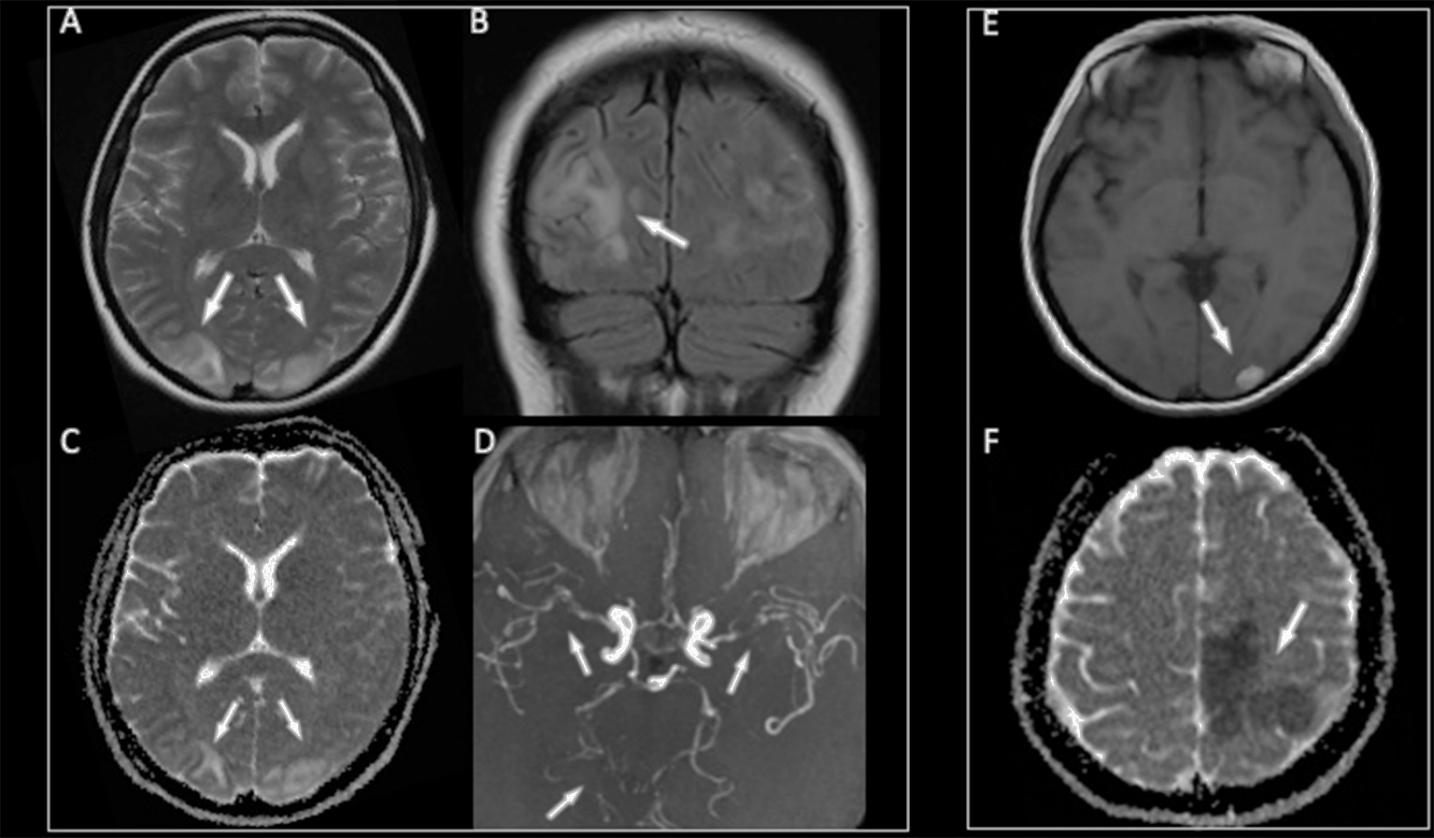
The MR examination showing symmetric bilateral cortical and subcortical hyperintensities on T2-weighted and fluid-attenuated inversion recovery (FLAIR) images **(A,B)** in the parieto-occipital lobes with a high signal in the same areas on the ADC map **(C)** due to vasogenic edema. The 3D-TOF MR angiography **(D)** demonstrating multifocal segmental areas of narrowing and dilatation of both the anterior and posterior arterial circulations. After 1 week, a hemorrhagic complication characterized by a bright signal on T1-weighted images **(E)** has appeared in the left occipital lobe, while a new ischemic lesion with a low signal on the ADC map **(F)** was noted in the frontal lobe.

The patient underwent magnetic resonance (MR) examination that showed the typical aspect of PRES lesions with bilateral, relatively symmetric, cortical, and subcortical parieto-occipital hyperintensities on T2-weighted and fluid-attenuated inversion recovery (FLAIR) images (Figures 1A,B), without diffusion restriction in the affected areas on the ADC map (Figure 1C) due to vasogenic edema. After Gadolinium administration, a mild and patchy enhancement, especially on the left side, was noted. An MR angiography with a 3D-time of flight (TOF) revealed multifocal segmental areas of narrowing and dilatation of both the anterior and posterior arterial cerebral circulation (Figure 1D).

The PRES was, therefore, suspected.

In the absence of autonomic dysfunction (no fluctuation of BP or HR was observed during continuous monitoring in the stroke unit), the immunomodulatory therapy was changed from IVIg to 5 sessions of plasma exchange (PEX) to arrest the rapid clinical worsening of a patient's conditions.

Due to the unfavorable course, 8 days after admission, a new MRI was performed, which revealed a hemorrhagic evolution of the lesion in the left occipital lobe (Figure 1E), associated with massive ischemic brain damage in the left frontal, temporal, and parietal lobes, and with the persistence of diffuse caliber reduction of all large vessels (Figure 1F).

A new diagnostic work-up was started; 11 days later, a CSF examination showed again marked albumin-cytological dissociation (2.3 gr/L with 5 lymphocytes at Nageotte); PCR for Varicella Zoster Virus (VZV) and Herpes simplex virus (HSV) 1–2, tick borne encephalitis (TBE) and Lyme serology, as well as standard microbiological cultures on cerebrospinal fluid (CSF), were negative.

Blood tests revealed an IgG title compatible with remote exposition to VZV, Citomegalovirus (CMV), Epstein Barr Virus (EBV), and Borrelia, while the IgM title was negative; screening for HIV, West Nile Virus (WNV), Hepatitis Viruses (A, B, C), and Toxoplasma was negative.

Autoimmune screening [antinuclear antibodies (ANA), extractable nuclear antigen (ENA), anti-neutrophil cytoplasmic antibodies (ANCA), complement C3, C4, cryoglobulins, antiphospholipid and anti-cardiolipin, Rheumatoid Factor, lupus anticoagulant] and systemic indices of inflammation [C-reactive protein (RCP) and Erythrocyte Sedimentation Rate (ESR)] were normal.

After PEX, the patient was treated with high-dose steroids (1-gr methylprednisolone for 2 days). Despite treatment, the evolution was unfavorable, and the patient needs to be transferred to the intensive care unit, where she died 15 days after the admission due to massive brain ischemia.

### Patient 2

A 59-year-old woman, with a history of mild hypertension and depression, treated with angiotensin-converting enzyme (ACE)-inhibitor and sertraline, arrived at our ER for symmetrical numbness and weakness in all limbs, which began 4 days earlier.

One month before, she had experienced fever for 3 days without other symptoms.

The neurological examination revealed flaccid paraparesis in the lower limbs and reduced muscle strength in the upper limbs; deep tendon reflexes were diffusely absent; distal deep sensitivity was abnormal. Cranial nerves were spared, and no sphincter dysfunction was present. On admission, her BP and HR were normal.

The CSF analysis showed an albumin-cytological dissociation with 0,4 cells and 1.1 gr/L of protein.

An extensive neurophysiological examination was performed, including motor nerve conduction studies, F-waves evaluation (median, SPE, and SPI nerves bilaterally and right ulnar nerve), and sensory nerve conduction studies (sural nerves bilaterally, right superficial radial, median and ulnar nerves, and left median nerve), which led to the diagnosis of an acute inflammatory demyelinating polyneuropathy (AIDP) variant of GBS (4).

Treatment with IVIg was started (0.4 g/Kg/die for 5 consecutive days). No fluctuations of BP or HR were reported during continuous monitoring of vital parameters in the stroke unit, and the patient did not experience any symptom of autonomic dysfunction.

The clinical status remained unchanged, but 5 days after IVIg therapy, she developed a bilateral loss of vision.

**Figure 2:**
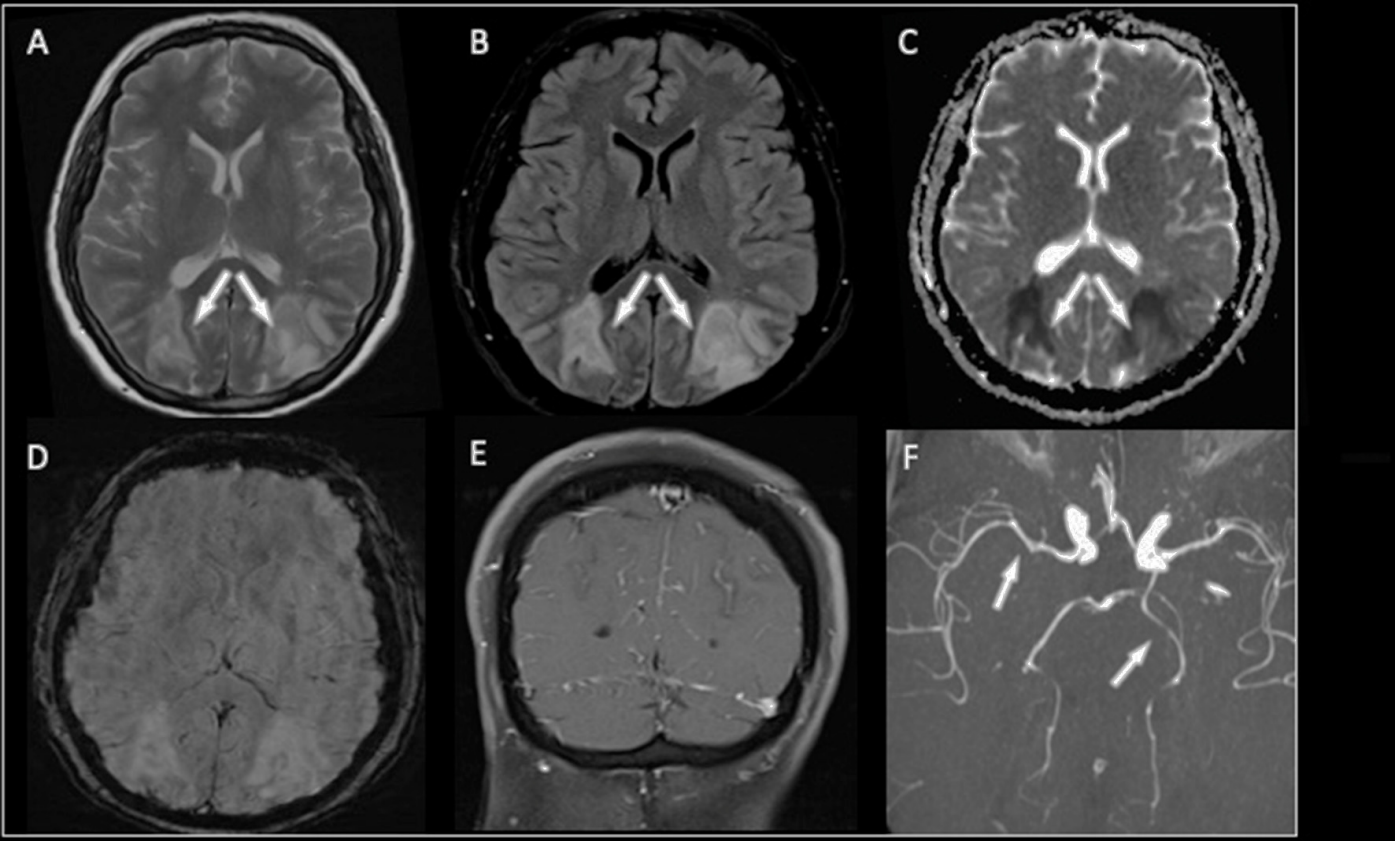
The MR examination demonstrated the presence of cortical and subcortical T2-weighted and FLAIR hyperintensities involving both parieto-occipital lobes **(A,B)**. In this case, the lesions were characterized by diffusion restriction on the ADC map **(C)** findings consistent with cytotoxic edema. No hemorrhagic transformation or calcifications on SWI were seen **(D)**. After contrast medium administration, diffuse leptomeningeal enhancement was noted **(E)**. The MR angiography with 3D-TOF sequence **(F)** showed slight narrowing of the P2 segment of the left posterior cerebral artery and the M1 segment of the right middle cerebral artery.

The patient underwent an MR examination that demonstrated the presence of cortical and subcortical T2 weighted and FLAIR hyperintensities with mild mass effect in both parietal lobes and the occipital horns of lateral ventricles (Figures 2A,B).

The lesions were characterized by diffusion restriction on the ADC map (Figure 2C), consistent with cytotoxic edema; no hemorrhage or calcification was present (Figure 2D). Diffuse leptomeningeal enhancement was noted after a contrast medium (Figure 2E), and MR angiography with a 3D-TOF sequence showed slight narrowing of a P2 segment of the left posterior cerebral artery (in this case, with fetal origin) of the basilar trunk and the M1 segment of the right middle cerebral artery (Figure 2F).

The PRES showing atypical imaging characteristics was, therefore, suspected.

Transcranial doppler ultrasound evaluation was compatible with cerebral vasospasm; mean flow velocity (MFV) in the right middle cerebral artery was 140 and 160 cm/s in the left one; the Lindegaard ratio was 4. The BP and HR values were normal during continuous monitoring. Cerebral vasospasm was treated with nimodipine, and, after 1 week, MFV was decreased to 65 cm/s bilaterally.

Due to the persistence of visual deficit and paraplegia, the patients underwent treatment with five sessions of PEX.

On clinical examination, performed 4 months after the discharge, the patient showed a mild improvement in motor function with residual moderate paresis in both legs; visual acuity was only partially recovered.

## Discussion

Although rare, the association between GBS and PRES has been previously described in the literature, with PRES preceding or following GBS diagnosis (5). Autonomic dysfunction with pressure changes, altered permeability of the blood-brain barrier in the central nervous system, and increased risk of encephalopathy after IVIg treatment are believed to be involved in the pathogenesis of PRES associated with GBS (6).

Up to date, about 30 cases of PRES associated with GBS have been reported (7). All but one of them demonstrated autonomic dysfunction, with high levels of BP [except for the case following Miller–Fisher syndrome (8)].

Even if IVIg therapy is believed to be associated with PRES, its pathogenetic role is questionable. Several cases of PRES are reported to occur before the administration of IVIg therapy (5). In addition, no case report of PRES has been reported after IVIg treatment performed for another disease aside from GBS or Miller–Fisher, except for a patient with end-stage renal failure and hematologic disease treated with chemotherapy, which are typical risk factors in developing PRES (9).

On the other side, a high prevalence of autoimmune disorders as risk factors in PRES has been identified. As reported by Pilato and colleagues, the presence of a systemic immune impairment should be considered in the pathogenesis of PRES, especially in normotensive patients; this possibility is supported by the fact that both our patients were suffering from GBS, which is strongly associated with a dysregulation of immune system response (3). In addition, the hypothesis of an altered endothelial function has been supposed, especially in patients with autoimmune diseases (10).

Moreover, the release of several cytokines in GBS (tumor necrosis factor-α, interleukin-6, interferon-γ, and IL-17) (11) is responsible for systemic immune activation, which, in our hypothesis, could lead to endothelial dysfunction and altered vascular permeability is seen in PRES.

As responsible for systemic inflammation, GBS should be considered a risk factor in the pathogenesis of PRES, independently of the presence of autonomic dysfunction.

Based on these considerations, we suggest the alternative possibility that PRES (and the coexistence of PRES-RCVS) might be caused by the altered endothelial function and by the immune system dysregulation that is present in GBS, supported by the fact that, in our patients, the continuous monitoring of vital parameters permitted the exclusion of hypertension, HR alteration, or other dysautonomic features that, if present, could have justified PRES.

Reversibility of the lesions and clinical aspects are a hallmark of PRES; however, several studies report a poor outcome, with permanent structural or clinical deficits, or even death in 26–37% and 8–19% of cases, respectively (12).

Up to 85% of patients presenting with PRES show some characteristics of RCVS-like cerebral vasoconstriction (13). We suggest the coexistence of RCVS syndrome both for some neuroradiological aspects (such as the segmental areas of narrowing and dilatation of several cerebral arteries) and for the poor clinical outcome, that it is reported to affect about 1/3 of patients with RCVS (3).

Even if vasoconstriction features could be demonstrated in some patients with PRES (14), the coexistence of these two syndromes has been previously reported. Nonetheless, more studies are needed to provide a more detailed description of this entity.

Due to the atypical clinical and neuroradiological features of our patients, the absence of BP alterations or other preexisting risks factors in intracerebral hemorrhage (such as hypertension or use of anticoagulants), as well as diffuse caliber reduction of all large cerebral vessels shown on MRI (first case) and an altered MFV, found on transcranial doppler ultrasound evaluation (second case), we hypothesize that PRES was associated with the simultaneous presence of a malignant RCVS-like cerebral vasoconstriction syndrome.

In literature, few cases are reporting an association between PRES and RCVS in patients with predisposing conditions (3), characterized by a more aggressive and sometimes unfavorable course, but no one after GBS.

In light of the prominent role of immune system activation and the systemic inflammatory status in GBS, we suggest the hypothesis of a central role of the immune system in the pathogenesis of PRES, or even of PRES/RCVS co-existing syndrome, and, subsequently, to endothelial dysfunction (15). This interpretation could justify the Gadolinium enhancement on MRI present in our two cases. The hypothesis of an altered endothelial function has been already proposed in previous studies, and it is believed to explain, at least partially, the pathogenesis of cytotoxic transformation and irreversibility of cerebral lesions seen in some atypical cases (3, 16).

Primary central nervous system (CNS) vasculitis is a rare but severe condition that affects cerebral and spinal cord vessels. For its clinical and neuroradiological characteristics, it is one of the main challenging differential diagnoses of RCVS. Calabrese and Mallek proposed diagnostic criteria for primary CNS vasculitis (17). Neuroradiological and angiographic imaging can increase the probability of a correct clinical diagnosis, although CNS biopsy remains mandatory to confirm the diagnosis of definite vasculitis.

In our two patients, vasculitis was considered among possible alternative diagnoses. However, the rapid onset of symptoms (headache, blurred vision, and altered mental status) in both the patient and the negativity of serological findings and cerebrospinal fluid analysis (except for mild elevation of proteins value, explained by GBS) is better explained by RCVS.

The MR angiography demonstrated areas of narrowing and dilatation of several cerebral arteries in both patients. Despite these findings, the differential diagnosis between vasculitis and RCVS cannot be done only on imaging data, without considering clinical and laboratory findings. This difficulty in distinguishing the two entities also remains for vessel-wall MRI, a new advanced technique to detect inflammation in the cerebral vessel (18).

## Conclusions

Our two cases show typical initial symptoms of PRES who successively developed permanent neurologic deficit and ischemia, leading to an unfavorable outcome.

We suggest that, in our cases, GBS may have generated a multifactorial condition of systemic inflammation, leading to endothelial dysfunction, in which insidious aspects, similar to RCVS, had complicated a typical clinical picture of PRES. In this scenario, GBS itself should be considered a risk factor in PRES/RCVS, independent of the presence of autonomic dysfunction. The exact mechanism underlining PRES/RCVS co-occurrence is still debated and crucial for therapeutic strategy. Although the role of IVIg treatment in the pathogenesis of PRES has been proposed, it remains debatable (5, 9). In conclusion, in the cases presented here, we highlight the hypothesis that autoimmune dysregulation, following GBS, and endothelial dysfunction could be responsible for the development of PRES/RCVS.

## Data Availability Statement

The original contributions presented in the study are included in the article/Supplementary Material, further inquiries can be directed to the corresponding author.

## Ethics Statement

Ethical review and approval was not required for the study on human participants in accordance with the local legislation and institutional requirements. The patients/participants provided their written informed consent to participate in this study. Written informed consent was obtained from the individual(s) for the publication of any potentially identifiable images or data included in this article.

## Author Contributions

EB, ID, SL, GM, and GG were involved in the management of the patients and drafting the manuscript. DB carried out the neuroradiological study. MV and GG were responsible for the project and the final revision of the text. All authors contributed to the article and approved the submitted version.

## Conflict of Interest

The authors declare that the research was conducted in the absence of any commercial or financial relationships that could be construed as a potential conflict of interest.

## Publisher's Note

All claims expressed in this article are solely those of the authors and do not necessarily represent those of their affiliated organizations, or those of the publisher, the editors and the reviewers. Any product that may be evaluated in this article, or claim that may be made by its manufacturer, is not guaranteed or endorsed by the publisher.

## Abbreviations

PRES: posterior reversible encephalopathy syndrome
RVCS: reversible cerebral vasoconstriction syndrome
GBS: Guillain-Barré syndrome
ER: emergency room
BP: blood pressure
HR: heart rate
AMAN: acute motor axonal neuropathy
IVIg: intravenous immunoglobulins
MR: magnetic resonance
FLAIR: fluid-attenuated inversion recovery
TOF: time of flight
PEX: plasma exchange
AIDP: acute inflammatory demyelinating polyneuropathy
MFV: mean flow velocity
CNS: central nervous system.

## References

[1] Fischer M Schmutzhard E. Posterior reversible encephalopathy syndrome. J Neurol. (2017) 264:1608–16. 10.1007/s00415-016-8377-8 28054130 PMC5533845

[2] Qubty W Irwin SL Fox CK. Review on the diagnosis and treatment of reversible cerebral vasoconstriction syndrome in children and adolescents. Semin Neurol. (2020) 40:294–302. 10.1055/s-0040-1702942 32079031

[3] Pilato F Distefano M Calandrelli R. Posterior reversible encephalopathy syndrome and reversible cerebral vasoconstriction syndrome: clinical and radiological considerations. Front Neurol. (2020) 11:34. 10.3389/fneur.2020.00034 32117007 PMC7033494

[4] Rajabally YA Durand MC Mitchell J Orlikowski D Nicolas G. Electrophysiological diagnosis of Guillain-Barré syndrome subtype: could a single study suffice?. J NeurolNeurosurg Psychiatry. (2015) 86:115–9. 10.1136/jnnp-2014-307815 24816419

[5] Rigamonti A Basso F Scaccabarozzi C Lauria G. Posterior reversible encephalopathy syndrome as the initial manifestation of Guillain-Barré syndrome: case report and review of the literature. J PeripherNerv Syst. (2012) 17:356–60. 10.1111/j.1529-8027.2012.00416.x 22971098

[6] Storti B Vedovello M Riva R Agazzi E Censori B Manara O. Posterior reversible encephalopathy and Guillain-Barré syndrome: which came first, the chicken or the egg? A review of literature. Neurol Sci. (2020) 41:3663–6. 10.1007/s10072-020-04496-1 32506357

[7] Salvalaggio A Cagnin A Marson P Ferracci F Cortelli P Corbetta M. Posterior reversible encephalopathy syndrome associated with Guillain-Barré syndrome: case report and clinical management considerations. J ClinApher. (2020) 35:231–3. 10.1002/jca.21783 32289176

[8] Ribeiro BN Salata TM Borges RS Marchiori E. Posterior reversible encephalopathy syndrome following immunoglobulin therapy in a patient with Miller-Fisher syndrome. Radiol Bras. (2016) 49:58–9. 10.1590/0100-3984.2015.0129 26929465 PMC4770401

[9] Belmouaz S Desport E Leroy F Teynie J Hannequin J Ayache RA. Posterior reversible encephalopathy induced by intravenous immunoglobulin. Nephrol Dial Transplant. (2008) 23:417–9. 10.1093/ndt/gfm594 17971381

[10] Fugate JE Claassen DO Cloft HJ Kallmes DF Kozak OS Rabinstein AA. Posterior reversible encephalopathy syndrome: associated clinical and radiologic findings. Mayo Clin Proc. (2010) 85:427–32. 10.4065/mcp.2009.0590 20435835 PMC2861971

[11] Sun T Chen X Shi S Liu Q Cheng Y. Peripheral blood and cerebrospinal fluid cytokine levels in guillainbarré syndrome: a systematic review and meta-analysis. Front Neurosci. (2019) 13:717. 10.3389/fnins.2019.00717 31379477 PMC6646663

[12] Siebert E Bohner G Liebig T Endres M Liman TG. Factors associated with fatal outcome in posterior reversible encephalopathy syndrome: a retrospective analysis of the Berlin PRES study. J Neurol. (2017) 264:237–42. 10.1007/s00415-016-8328-4 27815684

[13] Miller TR Shivashankar R Mossa-Basha M Gandhi D. Reversible cerebral vasoconstriction syndrome, part 1: epidemiology, pathogenesis, clinical course. AJNR Am J Neuroradiol. (2015) 36:1392–9. 10.3174/ajnr.A4214 25593203 PMC7964694

[14] Ducros A. Reversible cerebral vasoconstriction syndrome. Lancet Neurol. (2012) 11:906–17. 10.1016/S1474-4422(12)70135-7 22995694

[15] Saad AF Chaudhari R Wintermark M. Imaging of atypical and complicated posterior reversible encephalopathy syndrome. Front Neurol. (2019) 10:964. 10.3389/fneur.2019.00964 31551919 PMC6738024

[16] Cruz-Flores S de Assis Aquino Gondim F Leira EC. Brainstem involvement in hypertensive encephalopathy: clinical and radiological findings. Neurology. (2004) 62:1417–9. 10.1212/01.WNL.0000120668.73677.5F 15111687

[17] Calabrese LH Mallek JA. Primary angiitis of the central nervous system. Report of 8 new cases, review of the literature, and proposal for diagnostic criteria. Medicine (Baltimore). (1988) 67:20–39. 10.1097/00005792-198801000-00002 3275856

[18] Salvarani C Brown RD Jr Hunder GG. Adult primary central nervous system vasculitis. Lancet. (2012) 380:767–77. 10.1016/S0140-6736(12)60069-5 22575778

