# Supplementary figures and images for: Posterior Reversible Encephalopathy Syndrome in Guillain-Barré Syndrome: Just a Problem of Immunoglobulins? Controversy From Two Atypical Case Reports
Source: Front Neurol. 2022 Apr 6;13:817295. doi: 10.3389/fneur.2022.817295 (PMC9020620; doi:10.3389/fneur.2022.817295)

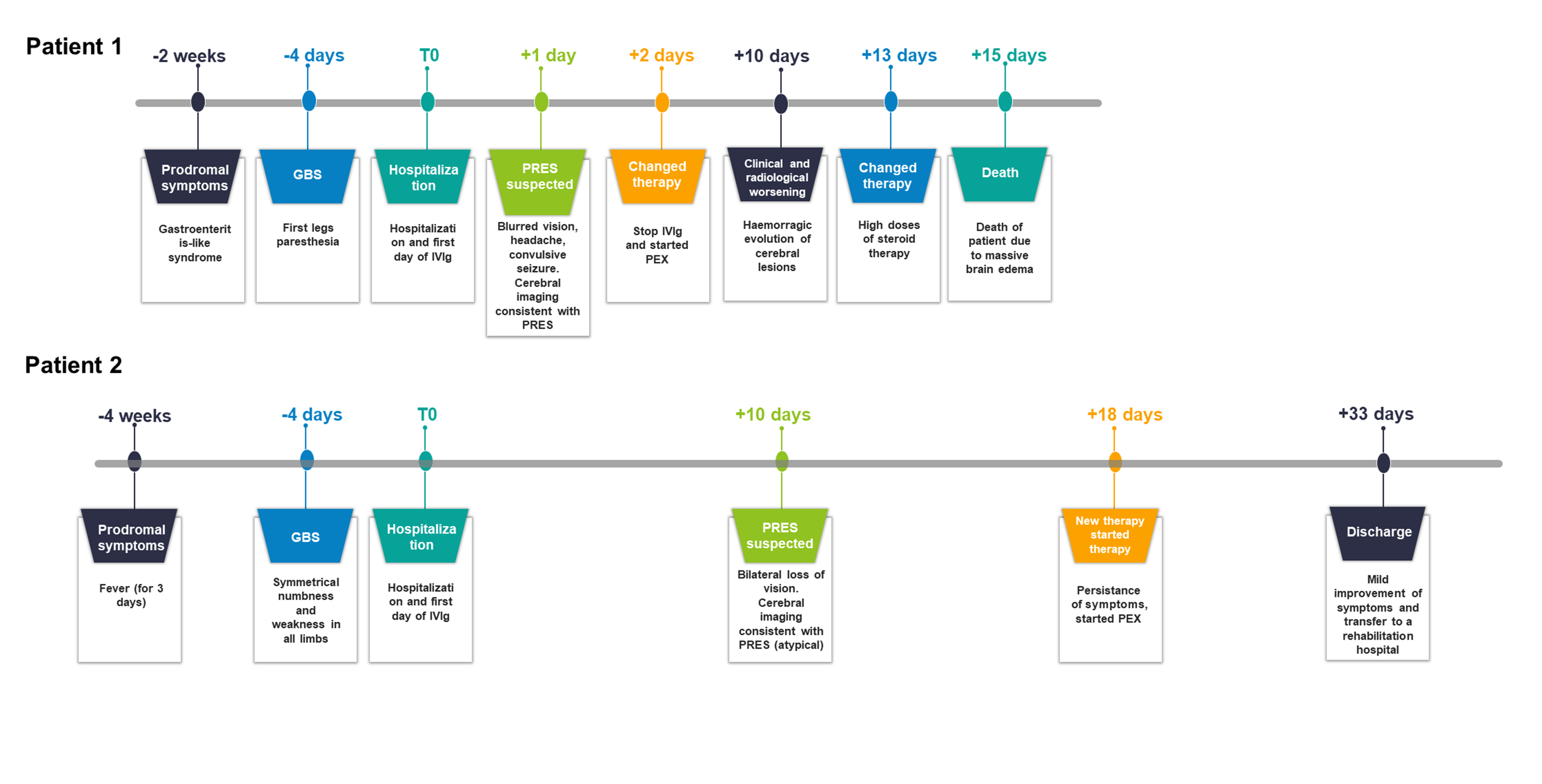

Supplement: Supplementary file 1 [file Image_1.TIF]
